# Functional Research on Three Presumed Asparagine Synthetase Family Members in Poplar

**DOI:** 10.3390/genes10050326

**Published:** 2019-04-28

**Authors:** Chunpu Qu, Bingqing Hao, Xiuyue Xu, Yuchen Wang, Chengjun Yang, Zhiru Xu, Guanjun Liu

**Affiliations:** 1 State Key Laboratory of Tree Genetics and Breeding (Northeast Forestry University), School of Forestry, Northeast Forestry University, Harbin 150040, China; qcp_0451@163.com (C.Q.); bqhao2019@163.com (B.H.); 15663593162@163.com (X.X.); abc1032788957@126.com (Y.W.); 2 School of Forestry, Northeast Forestry University, Harbin 150040, China; nxyycj@sina.cn; 3 Guangxi Forestry Research Institute, Nanning 530000, China; 4 College of Life Science, Northeast Forestry University, Harbin 150040, China

**Keywords:** poplar, asparagine synthetase, functional research, expression patterns, transcriptional regulation

## Abstract

Asparagine synthetase (AS), a key enzyme in plant nitrogen metabolism, plays an important role in plant nitrogen assimilation and distribution. Asparagine (Asn), the product of asparagine synthetase, is one of the main compounds responsible for organic nitrogen transport and storage in plants. In this study, we performed complementation experiments using an Asn-deficient *Escherichia coli* strain to demonstrate that three putative asparagine synthetase family members in poplar (*Populus simonii × P. nigra*) function in Asn synthesis. Quantitative real-time PCR revealed that the three members had high expression levels in different tissues of poplar and were regulated by exogenous nitrogen. *PnAS1* and *PnAS2* were also affected by diurnal rhythm. Long-term dark treatment resulted in a significant increase in *PnAS1* and *PnAS3* expression levels. Under long-term light conditions, however, *PnAS2* expression decreased significantly in the intermediate region of leaves. Exogenous application of ammonium nitrogen, glutamine, and a glutamine synthetase inhibitor revealed that *PnAS3* was more sensitive to exogenous glutamine, while *PnAS1* and *PnAS2* were more susceptible to exogenous ammonium nitrogen. Our results suggest that the various members of the *PnAS* gene family have distinct roles in different tissues and are regulated in different ways.

## 1. Introduction

Asparagine synthetase (*AS*; EC 6.3.5.4) is a chiefly cytoplasmic enzyme that generates asparagine from aspartate. This enzyme uses glutamine or ammonium as substrates to transfer amide groups to aspartic acid to form asparagine [1]. Asparagine (Asn), an important nitrogen transport compound, plays a major role in nitrogen utilization in new plant tissues [2,3,4,5,6,7]. AS is thus a key enzyme in nitrogen assimilation in higher plants.

The amino acid sequence of asparagine synthetase contains two highly similar conserved domains: the glutamine-amide transfer domain and the C-terminal synthetase domain [8]. The glutamine aminotransferase domain binds to glutamine (Gln). This domain, which extends from the N-terminus to the fourth amino acid position, has the structural characteristics of Met-Cys-Gly-Ile [9,10,11]. The synthetase domain includes three conserved sites, Cys, His, and Asp, which are localized to the N-terminal of the polypeptide [9,12]. The domains participate in the transamination of Gln [12]. In addition, aspartic acid- and adenosine monophosphate (AMP)-binding sites are located at the C-terminal of the polypeptide sequence [13]. At present, the gene encoding AS has been cloned from various plant species. The polypeptide encoded by *AS* generally contains 579–591 amino acids with a molecular weight of approximately 65 kDa [14]. 

According to phylogenetic analysis, the *AS* gene family can be divided into two subfamilies: class I and class II. Class I *AS* genes are usually inhibited by light. In common bean (*Phaseolus vulgaris*) and sunflower (*Helianthus annuus*), for example, high light inhibits the expression of these genes [15,16], possibly as the result of interaction with the photosynthesis process. In contrast, the expression profiles of class-II *AS* genes are relatively complex, and many are unrelated to light regulation [17,18,19]. In other studies, *AS* gene expression has been found to increase upon application of exogenous carbon [20,21,22].

AS is an important component of the nitrogen assimilation pathway. In previous studies, the expression level of the gene encoding the enzyme has been found to be related to nitrogen form and content. For example, *Arabidopsis thaliana AtAS2* is induced by NH_4_^+^, which increases its expression level [23]. As another example, *Phaseolus vulgaris PvAS1* and *PvAS2* [21] and soybean *SAS1, SAS2,* and *SAS3* genes are induced by NO_3_^−^ [3]. In poplar (*Populus* L.), the product of the AS enzyme, Asn, is a major nitrogen transport compound that plays a role in transporting nitrogen between sink and source tissues [24]. No reports have appeared on the composition of this gene family in poplar, however, and the pattern of differential expression and the mode of regulation of *AS* members in this plant species has not been studied.

*Populus simonii × P. nigra*, a hybrid poplar derived by crossing *P. pseudo-simonii* as the female parent with *P. nigra* as the male parent, is the main broad-leaved tree species in northeastern China and a model woody plant [25]. Nitrogen, a major element, must be absorbed from the external environment. In natural forest soils, nitrogen deficiency is often considered to be one of the main factors limiting tree growth [24]. Increasing the efficiency of nitrogen assimilation should thus significantly improve the material properties of poplar. To lay a theoretical foundation for research on poplar nitrogen assimilation and utilization, we identified members of the asparagine synthetase gene family and analyzed their spatial distribution in *Populus simonii × P. nigra*.

## 2. Materials and Methods 

### 2.1. Plant Material, Growth Environment and Sampling Method

In this study, we used approximately 15-cm-high seedlings of a single clone of *Populus simonii × P. nigra*. The seedlings were cultured in vermiculite. The nutrient solution was modified MS mediumwith change the NH_4_NO_3_ to 1 mM, which was replaced every 2 days. The seedlings were maintained for 30 days in a light-culture incubator under the following conditions: light intensity of 300 μmol m^−2^ s^−1^, 16-h/8-h light/dark photoperiod, the light phrase is 8:00–24:00, temperature of 24 ± 2 °C, and an air humidity of 40 ± 2%. Roots, stems, and various leaf portions of the poplar seedlings were selected for sampling. To study the expression patterns of *PnAS* genes in different regions of poplar leaves, we separated the leaves into three groups: the apical bud region (L1), comprising 1st, 2nd, and 3rd apical leaves; the intermediate region (L2), corresponding to 4th, 5th, and 6th leaves; and the more basal region (L3), including 9th, 10th, 11th, and 12th leaves. Following collection samples at 15:00. In the continuous light/dark experiment, all seedlings experienced 48 h of light/darkness, and other conditions were the same as those described above, correspondingly, the control sample is a group of seedlings that normally undergo 16-h light/8-h dark photoperiod. Samples were first rinsed with deionized water and then dried with absorbent paper. The samples were immediately frozen in liquid nitrogen and placed in a −80 °C freezer.

### 2.2. Search and Analysis of Gene Families

The poplar asparagine synthetase amino acid sequence was downloaded from Phytozome 12.0 (https://phytozome.jgi.doe.gov/pz/portal.html). AS amino acid sequences of other species, including *Arabidopsis thaliana, Escherichia coli, Helianthus annuus, Vitis vinifera, Populus trichocarpa, Astragalus sinicus, Vicia faba, Lotus japonicus, Glycine max, Phaseolus vulgaris,* and *Sorghum bicolor*, were downloaded from NCBI. A phylogenetic tree of the aligned sequences was constructed by the neighbor-joining method in MEGA5 [26].

### 2.3. *Escherichia coli* Complementation Experiment

The Asn auxotroph ER#4813 strain (asnB32, λ-, relA1, spoT1, asnA31, thi1), provided by the *E. coli* Genetic Stock Center (New Haven, CT, USA), was used for complementation studies. The coding region of *PnAS* was cloned into a *pET-14b* expression vector at the XhoI–NotI double-digestion site. Primers are listed in Table 1. Colonies carrying *pET14b, pET14b-PnAS1, pET14b-PnAS2*, or *pET14b-PnAS3* were cultured on solid agar M9 medium supplemented with a 1:100 dilution of 100 mg L^−1^ ampicillin and 0.1 mM IPTG. The optical density of the culture was measured at 600 nm after 3, 6, 9, 12, 24, 36, and 48 h of growth.

### 2.4. Nitrogen Treatment

Poplar seedlings cultured in vermiculite were supplied with nitrogen-free nutrient solution for 3 days and then subjected to one of the following treatments for 3 h, 12 h, or 72 h: 10 mM NH_4_NO_3_, 1 mM NH_4_NO_3_, 0.1 mM NH_4_NO_3_, 10 mM NH_4_Cl, 1 mM NH_4_Cl, 0.1 mM NH_4_Cl, 10 mM NaNO_3_, 1 mM NaNO_3_, or 0.1 mM NaNO_3_. Seedlings not supplied with nitrogen nutrient solution were treated as controls. To avoid the effects of diurnal rhythm, all samples were collected at the same time.

### 2.5. RNA Extraction and Quantitative Real-Time PCR Detection

Total RNA was extracted from approximately 100 mg of plant tissue using pBIOZOL Total RNA extraction reagent (BioFlux, Tokyo, Japan) according to the manufacturer’s instructions. Extracted RNA (1 µg) was treated with RNase-free DNase I and then used for single-strand cDNA synthesis with a reverse transcription kit (SYBR Premix Ex Taq; Takara). Real-time PCR was carried out according to a SYBR Green fluorescence-based procedure using UltraSYBR Mixture reagents (CWBIO, Beijing, China). The PCR cycling protocol consisted of an initial denaturation at 95 °C for 10 min, followed by 45 cycles of 95 °C for 15 s and 60 °C for 1 min. After the final cycle, a melting curve analysis was performed over a temperature range of 60–95 °C in increments of 1 °C to verify the reaction specificity. Using the actin gene [27] as a constitutive reference, relative expression was measured by the 2^−ΔΔCt^ method [28]. The primers used in this study are given in Table 2.

### 2.6. Statistical Analysis

At the time of material extraction, three seedlings were mixed for treatment under the same treatment conditions, and three replicates, each composed of one group of mixed samples, were used for subsequent experiments. Values were calculated as means ± SD. Statistical analyses were performed using SPSS Statistics software (version 20). Data were compared by multivariate analysis followed by Duncan’s multiple range test, with a value of *p* ≤ 0.05 used as the criterion indicating significant differences between studied conditions.

## 3. Results

### 3.1. Sequence Analysis of Asparagine Synthetase Family Members in *Populus simonii* × *P. nigra*

By searching the poplar Phytozome database, we obtained three putative asparagine synthetase family members with gene IDs Potri.009G072900, Potri.005G075700, and Potri.001G278400, respectively. Primers were designed for the above three gene sequences, and three full-length coding sequences, designated as *PnAS1, PnAS2*, and *PnAS3*, were successfully obtained from the *Populus simonii × P. nigra* cDNA library (Table 3). Similarities between *PnAS1–3* and the homologous *Populus trichocarpa* sequences were 99%, 99%, and 98%, respectively. Their putative ORF regions comprised 1770, 1755, and 1764 bp, and predicted protein molecular weights were 66, 65.6, and 65.8 KDa. PnAS1–3 proteins and nucleic acids shared 68–95% identity (Table 4).

**Figure 1:**
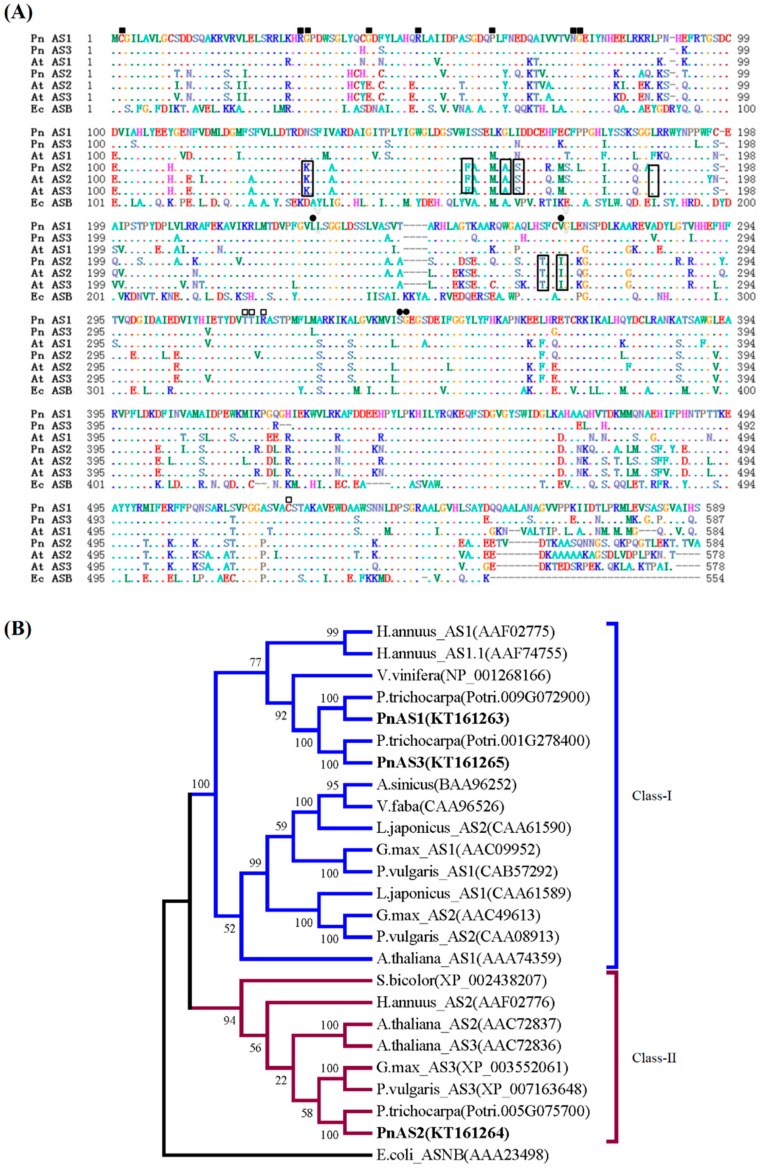
Sequence and phylogenetic analyses of poplar PnAS proteins. (**A**) Alignment between PnAS1–3, Arabidopsis AtAS1–3, and *Escherichia coli* EcASNB protein sequences. Dots indicate identical amino acids, and dashes represent gaps introduced to maximize similarity. Essential residues of glutamine- and aspartate-binding domains are denoted by solid and open squares. Solid circles indicate residues involved in the anchoring of the AMP moiety, and residues in the pyrophosphate-binding area are marked with open circles. Residues conserved in all class-II AS proteins are boxed. (**B**) Phylogenetic tree of asparagine synthetase sequences from *Populus simonii × P. nigra*, *Arabidopsis thaliana, Escherichia coli, Helianthus annuus, Vitis vinifera, Prunus trichocarpa, Astragalus sinicus, Vicia faba, Lotus japonicus, Glycine max, Phaseolus vulgaris,* and *Sorghum bicolor*. The tree was generated from the aligned sequences by the neighbor-joining method in MEGA5.

As shown in Figure 1, the three *PnAS* genes had glutamine-binding domain (GAT) residues (Met-Cys-Gly-Ile), residues corresponding to the synthetase domain (Cys2, Asp34, and His104), and aspartate-binding sites (Thr315, Thr316, Arg318, and Cys520). Another residue binding to pyrophosphate (between Ser232 and Ser237) and residues necessary for binding to the AMP portion of ATP (Leu230, Ile266, Ser340, and Gly341) [13] were also found to be conserved in all PnAS proteins. According to the results of phylogenetic analysis, PnAS1 and PnAS3 belong to class I, and PnAS2 belongs to class II.

### 3.2. Asn-Deficient *Escherichia coli* Complementation Experiment

**Figure 2:**
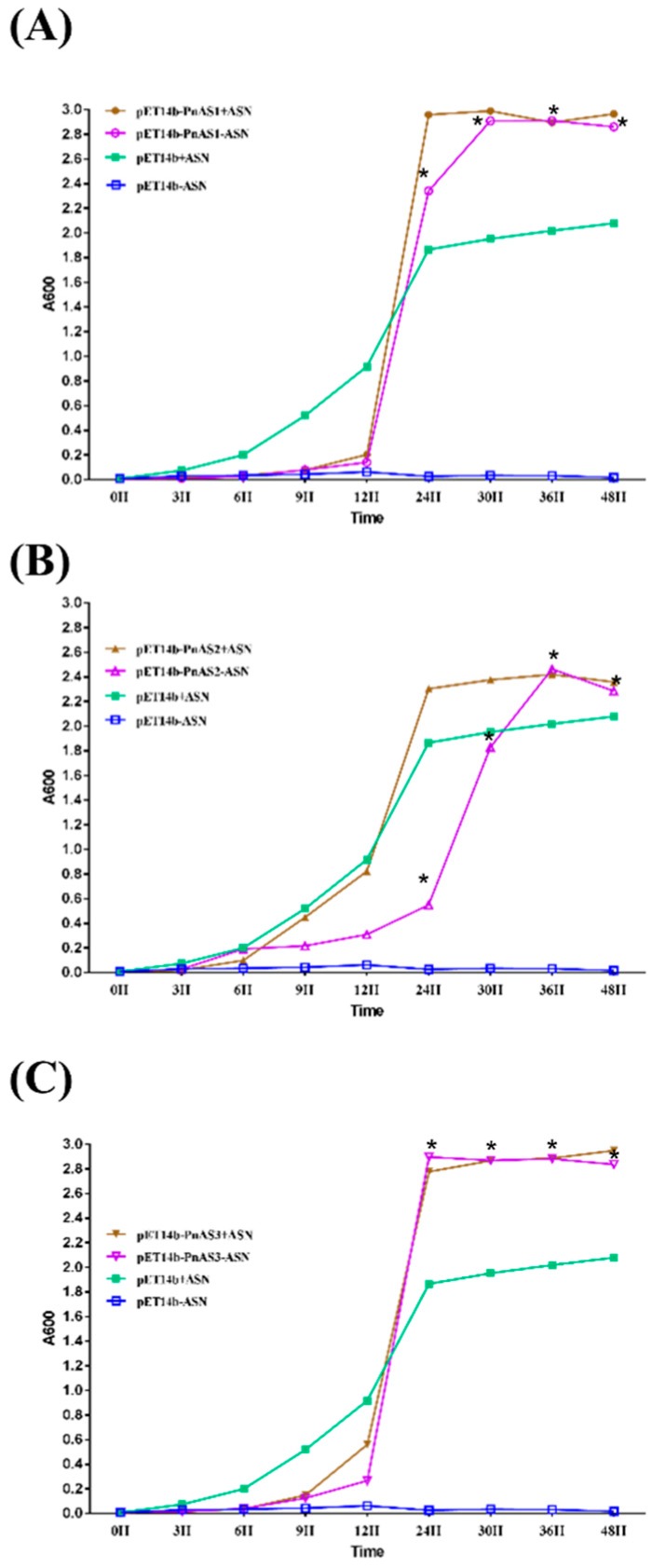
Asn-deficient *E. coli* complementation experiment. Transformed ER strains harboring *pET-14b* (**A**–**C**), *pET-14b-PnAS1* (**A**), *pET-14b-PnAS2* (**B**), or *pET-14b-PnAS3* (**C**) were grown in Asn-free and Asn-containing media, and OD values were measured at 0, 3, 6, 9, 12, 24, 30, 36, and 48 h. The asterisk indicates a significant difference in the OD values between the *pET-14b-ASN* and *pET-14b-PnAS1/2/3-ASN* groups (*p* ≤ 0.05).

The newly constructed prokaryotic expression vectors *pET-14b, pET-14b-PnAS1, pET-14b-PnAS2*, and *pET-14b-PnAS3* were transformed into an *E. coli* auxotrophic ER strain lacking AS activity (asnA, asnB, thi-1, relA, spoT1) [29]. In medium lacking Asn, the growth of *E. coli* ER transformed with the empty vector was very weak, and the growth curve barely changed. In contrast, the *E. coli* auxotrophic ER strain transformed with *pET-14b-PnAS1, pET-14b-PnAS3*, or *pET-14b-PnAS2* could be grown in M9 medium without Asn and exhibited an S-type growth curve. These results confirm that all three asparagine syntheases can significantly improve the growth ability of *E. coli*. (Figure 2).

The *E. coli* auxotrophic ER strain transformed with *pET-14b-PnAS3* had the highest OD value at 24 h, while the ER strains transformed with *pET-14b-PnAS1* or *pET-14b-PnAS2* reached their maximum growth at 30 h and 36 h, respectively. These results indicate that *PnAS1*, *PnAS2*, and *PnAS3* genes play roles in the synthesis of asparagine synthetase and maintained the growth of the *E. coli* auxotrophic ER strain in the absence of Asn. In addition, the recovery ability of *PnAS3* was slightly higher than that of *PnAS1* and *PnAS2* (Figure 2).

### 3.3. Spatiotemporal Specific Expression Patterns of the Three AS Genes in *Populus simonii* × *P. nigra*

**Figure 3:**
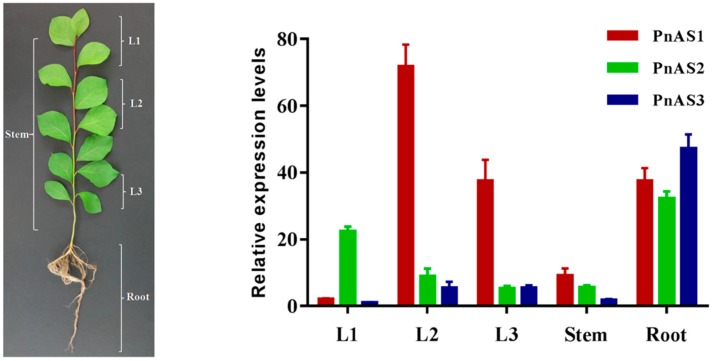
Relative expression levels of *PnAS1, PnAS2*, and *PnAS3* in different organs of poplar seedlings. The real-time qRT-PCR analysis was performed on total RNA samples extracted from roots (R), stems (S), and leaves from different plant regions, namely, apical bud (L1), intermediate (L2), and more basal (L3) regions. *PnAS1* expression levels in L1 were referred to show relative expression.

We studied the expression patterns of the three asparagine synthetase genes in *Populus simonii × P. nigra* by real-time PCR. According to the results, the three genes were expressed in roots, stems, and leaves, but their expression levels were not consistent. *PnAS1* gene expression was particularly high in leaves (L2 and L3), especially in L2 leaves, whereas *PnAS2* was highly expressed in L1 leaves and in roots. The expression of *PnAS3* was low in all tissues except roots (Figure 3).

**Figure 4:**
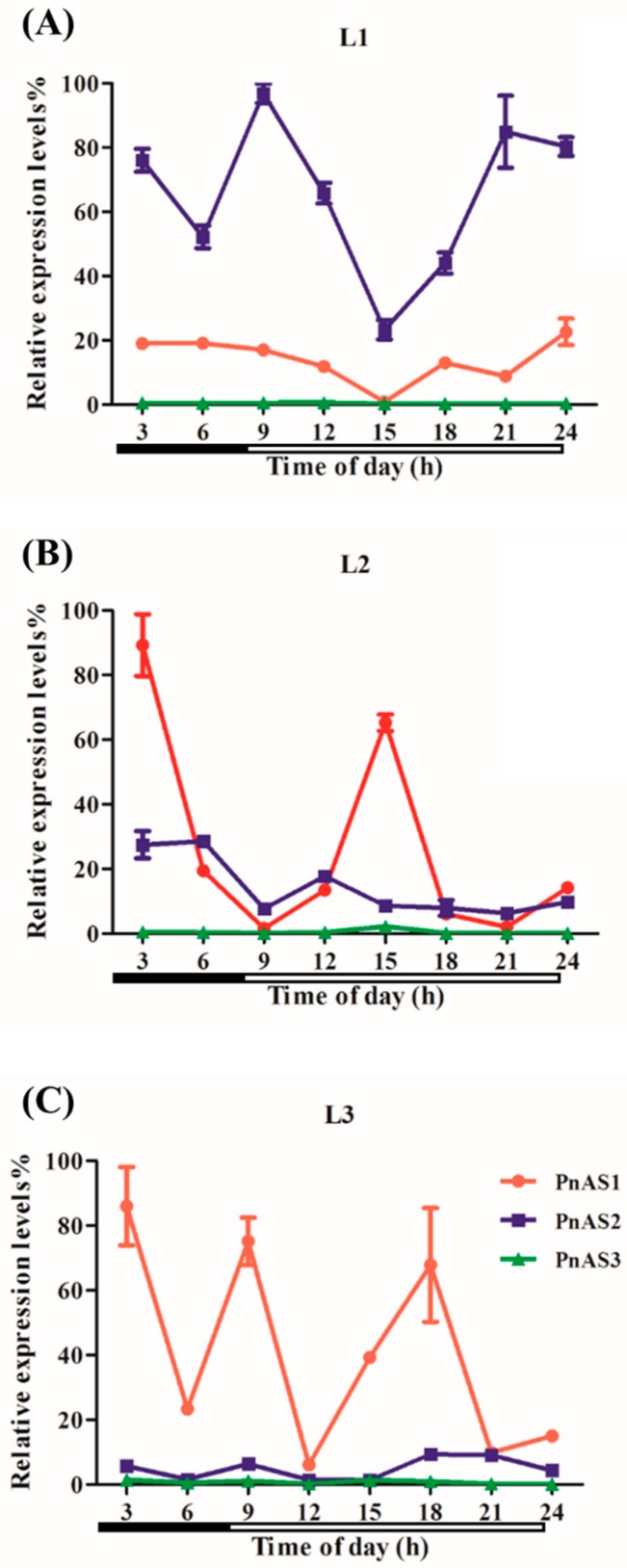
Changes in expression patterns of three *AS* genes at (**A**) L1, (**B**) L2, (**C**) L3 leaf positions in response to diurnal rhythm. White bars and black bars represent light and dark phrase, respectively.

The effect of diurnal rhythm on the above three genes is shown in Figure 4. In general, the expression levels of *PnAS1* and *PnAS2* genes varied with time. In L1 leaves, the expression level of *PnAS2* was higher than that of *PnAS1*. In L2 leaves, the expression level of *PnAS2* was basically the same as that of *PnAS1*, except around 3:00 and 15:00. In L3 leaves, the expression of *PnAS1* fluctuated, and *PnAS2* expression was lower than that of *PnAS1*. *PnAS3* expression levels were low in leaves from the three different positions and were apparently unaffected by diurnal rhythm, consistent with the weak expression of *PnAS3* observed in shoots.

**Figure 5:**
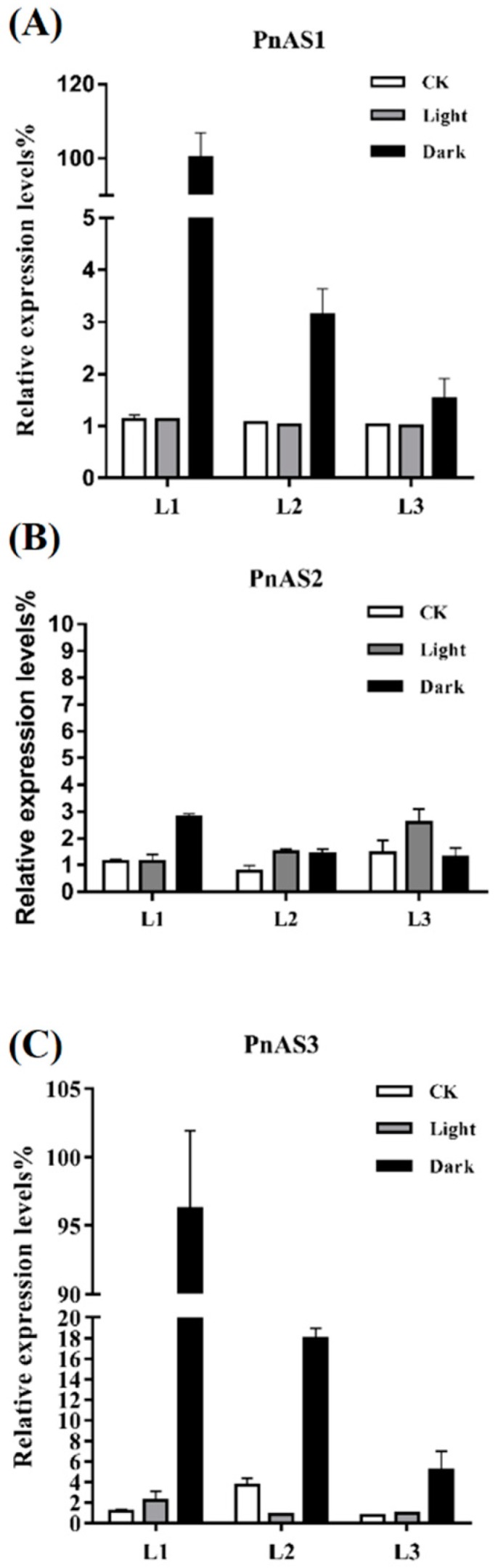
Relative expression levels of *PnAS1* (**A**), *PnAS2* (**B**), and *PnAS3* (**C**) under continuous light/dark conditions. The control (CK) reflects expression under a normal photoperiod.

At the transcriptional level, the three genes exhibited significant changes under continuous light or dark treatments (Figure 5). In the case of continuous light, the expression levels of *PnAS1* at the three different leaf positions were not significantly different from that under a normal photoperiod, but increased significantly under dark treatment. In particular, *PnAS1* expression at the L1 leaf position exhibited the greatest increase under dark treatment, followed by L2 and then L3. The expression pattern of *PnAS3* was very similar to that of *PnAS1*, but the expression level of *PnAS2* seemed to be unrelated to conditions such as long-term illumination or dark treatment.

### 3.4. The Effect of Exogenous Nitrogen on Patterns of Asparagine Synthetase Gene Expression

**Figure 6:**
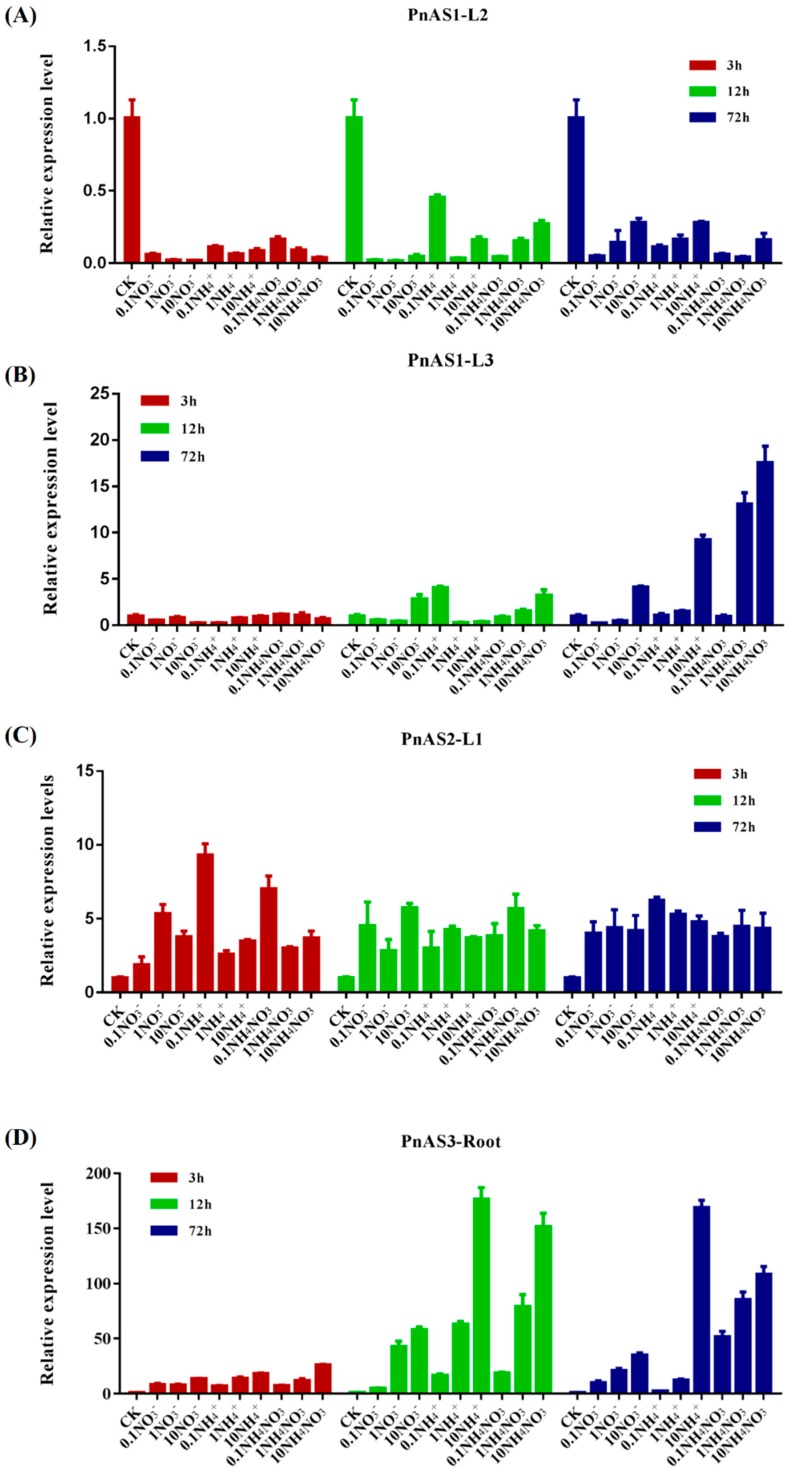
Changes in expression patterns of three *AS* genes in response to different forms and concentrations of nitrogen. (**A**–**D**) Changes in expression patterns of the *PnAS1* gene at the L2 leaf position (**A**), the *PnAS1* gene at the L3 leaf position (**B**), the *PnAS2* gene at the leaf L1 position (**C**), and the *PnAS3* gene in roots (**D**).

Asparagine synthetase is closely related to nitrogen assimilation. We therefore treated *Populus simonii × P. nigra* with different nitrogen forms (ammonium or nitrate) and concentrations and studied the induction of the three asparagine synthetase genes. The results are shown in Figure 6.

Relative changes in *PnAS1* expression in response to different nitrogen forms and concentrations are displayed in Figure 6A,B. As shown in the figure, *PnAS1* expression was significantly decreased at the L2 position under treatment; in L3 leaves, in contrast, high concentrations of nitrogen (>1 mM) for 72 h increased the expression of *PnAS1*. As can be seen in Figure 6C, which shows the relative change in *PnAS2* expression under different nitrogen forms and concentrations, the gene was induced in L1 leaves by all nitrogen forms and concentrations. Figure 6D shows the relative change in *PnAS3* expression under different forms and concentrations of nitrogen; the gene was strongly induced by nitrogen in roots and its expression level was increased, with its expression increasing as the nitrogen treatment was prolonged. These observations indicate that the three members respond differently to external nitrogen treatment, a result possibly related to their different functions.

### 3.5. Expression Trend of PnAS under MSX Treatment

**Figure 7:**
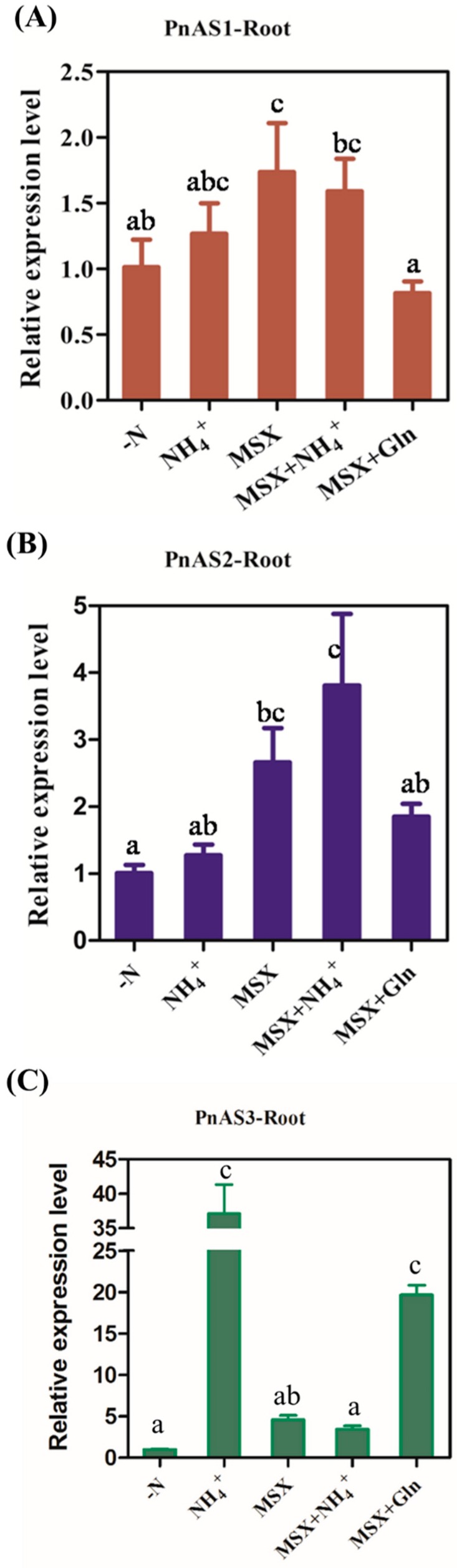
Expression trends of *PnAS1* (**A**), *PnAS2* (**B**), and *PnAS3* (**C**) in roots after treatment with -N, NH_4_^+^, MSX, MSX+NH_4_^+^, or MSX+Gln.

As shown in Figure 7, the *PnAS3* gene was strongly induced by NH_4_^+^, and its expression level increased. After addition of the glutamine synthetase inhibitor MSX, the expression level of the *PnAS3* gene was significantly lower than that observed after addition of NH_4_^+^, and its expression could not be restored by exogenous application of NH_4_^+^. After the addition of Gln, however, *PnAS3* gene expression was still at a high level even in the presence of MSX, which indicates that the expression of *PnAS3* is regulated by Gln or Gln derivatives, and the *PnAS1* and *PnAS2* genes expression level were only slightly increased by NH_4_^+^.

## 4. Discussion

Asparagine synthetase, an aminotransferase encoded by a small gene family, is widely present in plants. Plant asparagine synthetases can be classified into two categories, namely Class I and Class II [16,17]. Asparagine synthetase uses NH_4_^+^ or Gln to synthesize Asn, which plays an important role in nitrogen metabolism and transportation and is one of the main compounds involved in nitrogen transport in plants. The transportation of asparagine allows the required amide nitrogen to be shuttled to different positions in plants [20,30].

Previous studies have found that exogenous nitrogen sources are also one of the main factors regulating the expression of *AS* genes [3,23,31]. For example, nitrate nitrogen can induce the expression of *PVAS1* and *PVAS2* in *Phaseolus vulgaris* [21] and *SAS1, SAS2*, and *SAS3* in soybean [3]. High concentrations of ammonium are more likely to up-regulate the barley *AS* gene [32]. In poplar, however, *AS* gene types and quantities, temporal and spatial expression patterns, and regulation methods are still unclear. In this study, we analyzed three putative poplar *PnAS* genes and determined that all three play a role in Asn synthesis. Phylogenetic analysis revealed that these three genes belong to the two major classes. According to an analysis of spatiotemporal expression patterns, *PnAS1, PnAS2*, and *PnAS3* had their highest expressions in old leaves, new leaves, and roots, respectively. *PnAS1* and *PnAS2* were affected by diurnal rhythm and light/darkness treatments. *PnAS1, PnAS2*, and *PnAS3* were found to be respectively up-regulated in old leaves, new leaves, and roots by exogenous nitrogen. An experiment involving an MSX inhibitor indicated that Gln can induce up-regulation of *PnAS3*. These experiments lay a foundation for further functional elucidation of poplar *AS* genes.

Asn is a major nitrogen transport compound in poplar and is important in the process of nitrogen transportation from roots to shoots [33,34,35]. In previous studies, the expression patterns of nitrogen-assimilation related genes were not consistent between different poplar leaf positions [36]. In this study, we therefore divided the leaves into three regions and investigated the differences of *PnAS1-3* expression characteristics in each region. We found that the expression levels of the three *PnAS* family members were not consistent in different tissues. As shown in Figure 3, *PnAS1, PnAS2*, and *PnAS3* had their highest expressions in old leaves, new leaves, and roots, respectively. Previous studies have shown that most *AS* gene family members in plants exhibit temporal- and spatial-specific expression that is closely related to the functions of these genes in different plant parts [21,37,38]. We therefore speculate that the three family members exert their respective roles in different parts of the plant. As revealed by the results of exogenous nitrogen treatment shown in Figure 6, different forms and concentrations of nitrogen can induce or inhibit high-expression gene family members in certain tissues, consistent with the results of previous studies [3,20,23,31], but the effect on other relatively lowly expressed genes in the same tissue is more complicated.

As shown in Figure 4, the effect of diurnal rhythm on the different *AS* gene members was not consistent. *PnAS2* had the highest expression in L1 leaves, but its expression fluctuated dramatically and decreased between 9:00 and 21:00. In L2 leaves, the expression level of *PnAS1* was higher than that of *PnAS2* around 3:00 and 15:00; in other time periods, however, the expression of *PnAS1* was similar to that of *PnAS2*. Previous studies have shown that *HAS1* and *HAS1.1* genes identified in *H. annuus* exhibit enhanced transcript expression under dark conditions that is inhibited by the addition of sucrose [20]. In *Arabidopsis thaliana*, light also inhibits the expression of *Asn1* mRNA, and sucrose can produce a similar inhibitory effect; plants may thus have some sort of mechanism to sense organic nitrogen and carbon to allow metabolic pathway regulation [19].

Because previous studies have shown that exogenous light and sucrose can cause changes in the expression levels of *AS1* and *AS2* [16,20], we examined changes in expression patterns of genes such as *PnAS1* and *PnAS2* under long-term light and dark conditions. We observed that *PnAS1* and *PnAS3* expressions were significantly increased under dark conditions, while *PnAS2* expression significantly increased only in L1 leaves under dark conditions (Figure 5). Asparagine and glutamine are the two major nitrogen transport compounds in plants [34,35]. Asparagine has a higher N/C ratio (2N:4C) than glutamine (2N:5C). Under dark conditions, plant photosynthesis stops and sugar content decreases; consequently, the use of asparagine for nitrogen transport is more economical compared with glutamine. *PnAS* genes are therefore induced by dark conditions, and their increased expressions may be related to the need to synthesize asparagine for plant nitrogen utilization under dark conditions.

Previous studies have shown that both NH_4_^+^ and Gln may act as signaling substances to regulate nitrogen assimilation-related genes [39,40]. In plants, NH_4_^+^ can be assimilated by glutamine synthetase to form Gln.Some members of the *AS* family are up-regulated by exogenous nitrogen. For example, Wong et al. found that Arabidopsis *AS2* is up-regulated by NH_4_^+^ [23], and a similar result has been observed for the *AS* gene in soybean [3]. After exogenous application of NH_4_^+^, the expression level of poplar *PnAS3* in roots was significantly increased in our study; after adding the glutamine synthetase inhibitor MSX, however, *PnAS3* relative expression was restored to a level close to that before the addition of nitrogen, and this was still true even after applying exogenous NH_4_^+^. Even if MSX was present, the expression level of *PnAS3* was also up-regulated after the addition of Gln, and its level was nearly the same as that observed when only NH_4_^+^ was applied.

*PnAS1* and *PnAS2* were also up-regulated by NH_4_^+^ in roots, but the expression levels of both were increased by the action of MSX. After adding Gln, however, their expression levels did not increase. We hypothesize that poplar *PnAS3* is up-regulated by Gln but not by NH_4_^+^, while *PnAS2* and *PnAS1* seem to be more inhibited by Gln. This result indicates that the regulatory patterns of different members of the family are also different. Exogenous Gln and NH_4_^+^ may control the expressions of different *AS* gene family members. The mode of regulation of expressions of the different members vary greatly. Previous studies have also shown that different *AS* genes have diverse responses to external conditions [16,41,42,43,44].

In summary, we have studied the functions of three presumed asparagine synthetase genes in poplar. We observed that the three putative asparagine synthetases had biological activities and high expression levels in leaves and roots, but their expression patterns in response to exogenous nitrogen were inconsistent. We hypothesize that these genes are regulated differently and that *PnAS3* expression is affected by Gln. Because the regulatory modes of these three genes are still unclear, we plan to explore the regulatory mechanisms in more detail and to further reveal the respective functions of poplar asparagine synthetases during nitrogen assimilation and mobilization.

## Figures and Tables

**Table 1:** Primers used for insertion of *PnAS* coding regions into the prokaryotic expression vector.

Name	Forward (5′–3′)	Reverse (5′–3′)
*PnAS1-HF*	CATGCCATGGGCATGTGTGGGATA	ATAGTTTAGCGGCCGCCTAACTGTG
*PnAS2-HF*	CCGCTCGAGATGTGCGGCATCCT	ATAGTTTAGCGGCCGCTCAAGCAACT
*PnAS3-HF*	CATGCCATGGGCATGTGTGGGATA	ATAGTTTAGCGGCCGCCTAACTTTG

**Table 2:** Primers used for real-time PCR.

Gene	Accession No	Forward (5′–3′)	Reverse (5′–3′)
*PnAS1*	KT161263	TGTTGGAAGTTAGTGCTTCGG	GACAACACACGACTTCAAAGGA
*PnAS2*	KT161264	ACCAAGGCTGCCAGTCAGAATAA	CGTACCCTAACTAAAGCGAACGAAA
*PnAS3*	KT161265	GAGGACCGAAGTACATGCC	CAACAAGGTGCCAACACTACT
Actin	XM_002298946	CACAACTGCTGAACGGGAAAT	CAGGGCAACGGAAACACTCT

**Table 3:** Primers used for cloning of *PnAS1–3*.

Name	Forward (5′–3′)	Reverse (5′–3′)
*PnAS1*	ATGTGTGGGATACTTGCTG	CTAACTGTGGATCGCAAC
*PnAS2*	ATGTGCGGCATCCTCGCTG	TCAAGCAACTGTTGCAGT
*PnAS3*	ATGTGTGGGATACTTGCTGTTT	CTAACTTTGGATTGCAACTCCTG

**Table 4:** Protein and nucleic acid sequence similarities between three distinct *Populus simonii × P. nigra* asparagine synthetases.

	PnAS1	PnAS3	PnAS2
**PnAS1**	—	*91%*	*68.80%*
**PnAS3**	**94%**	—	*69.50%*
**PnAS2**	**80%**	**77.80%**	—

Note: Protein similarities (left side of matrix, bold font) and nucleic acid similarities (right side of matrix, italics) were calculated with NCBI BLAST.
